# Osteology and phylogeny of *Panzhousaurus rotundirostris* from the Middle Triassic (Anisian) of Guizhou Province, China, and implications for the origin of Keichousauridae (Pachypleurosauroidea, Eosauropterygia)

**DOI:** 10.7717/peerj.21267

**Published:** 2026-05-07

**Authors:** Feng-Ting Tan, Guang-Hui Xu, Qing-Hua Shang

**Affiliations:** 1 Key Laboratory of Vertebrate Evolution and Human Origins, Institute of Vertebrate Paleontology and Paleoanthropology, Chinese Academy of Sciences, Beijing, China; 2 College of Earth and Planetary Sciences, University of Chinese Academy of Sciences, Beijing, China

**Keywords:** South China, Triassic, Marine reptiles, Morphology, Taxonomy

## Abstract

Sauropterygia (including Placodontia and Eosauropterygia) is the most species-rich group of marine reptiles in the Mesozoic. *Panzhousaurus rotundirostris* is a rare eosauropterygian from the early Middle Triassic (Anisian) marine deposits of Panzhou, Guizhou Province, China. Until recently, *P. rotundirostris* has been known only by two specimens, and its phylogenetic position within Eosauropterygia remains much controversial. A taxonomic revision of *P. rotundirostrisis* is provided here based on a comparative study of the type material and a new, juvenile specimen from the same locality and horizon as the holotype. The revised description accommodates significant changes to the reconstruction of *P. rotundirostris*, including the skull roof, circumorbital bones, and jaws. The juvenile status of the new specimen is well confirmed by a series of features (*e.g.*, smaller body size, relatively larger orbit, unfused neural arches, and fewer carpal ossifications and caudal ribs). The wealth of new anatomical data for this species has facilitated a phylogenetic analysis, the results of which recover *Panzhousaurus* as a keichousaurid and favour the sister group relationships between Keichousauridae and Pachypleurosauridae. The topology provides new insights into the origin and evolution of Keichousauridae, a major clade of eosauropterygian marine reptiles endemic in the Middle Triassic of China.

## Introduction

Following the end-Permian mass extinction (∼252 Ma), some reptiles (*e.g*., Ichthyosauria, Sauropterygia and Thalattosauria) invaded marine ecosystems and occupied various trophic levels through the Mesozoic (Merriam, 1910; Motani, 2009; Bardet et al., 2014; Benton & Wu, 2022). Among them, Sauropterygia (including Placodontia and Eosauropterygia) is the most diverse lineage that has a rich fossil record from the Early Triassic Olenekian to the Late Cretaceous (Rieppel, 2000; Neenan, Klein & Scheyer, 2013; Neenan et al., 2017; Jiang et al., 2014; Wang et al., 2022). Pachypleurosauroidea is an early-diverging group of eosauropterygians, comprising pachypleurosaurids, keichousaurids and closely related taxa (Carroll & Gaskill, 1985; Sander, 1989; Rieppel & Lin, 1995; Liu et al., 2011; Shang & Li, 2015; Xu et al., 2022; Xu et al., 2023; Hu, Li & Liu, 2024). Until recently, 18 genera have been referred to the Pachypleurosauroidea, including seven from the Middle Triassic of Europe (Sues & Carroll, 1985; Rieppel, 1989; Sander, 1989; Klein, 2009; Renesto, Binelli & Hagdorn, 2014; Klein & Surmik, 2021; Klein et al., 2022) and 11 from the Early to Middle Triassic of South China (Young, 1958; Jiang et al., 2008a; Jiang et al., 2008b; Jiang et al., 2008c; Jiang et al., 2014; Jiang et al., 2019; Liu et al., 2011; Shang, Wu & Li, 2011; Shang, Wu & Li, 2017; Shang & Li, 2015; Cheng et al., 2016; Xu et al., 2022; Xu et al., 2023). Because the pachypleurosauroids retain many plesiomorphic characteristics for sauropterygians, they are critical for investigating the origin and early evolution of this clade. However, the relationships of pachypleurosauroids have remained controversial, particularly concerning the placements of some genera (*e.g*., *Keichousaurus*, *Panzhousaurus* and *Wumengosaurus*) from the Middle Triassic of Guizhou Province, China (Holmes, Cheng & Wu, 2008; Jiang et al., 2008a; Jiang et al., 2008b; Jiang et al., 2008c; Jiang et al., 2019; Wu et al., 2011; Xu et al., 2023).

**Figure 1:**
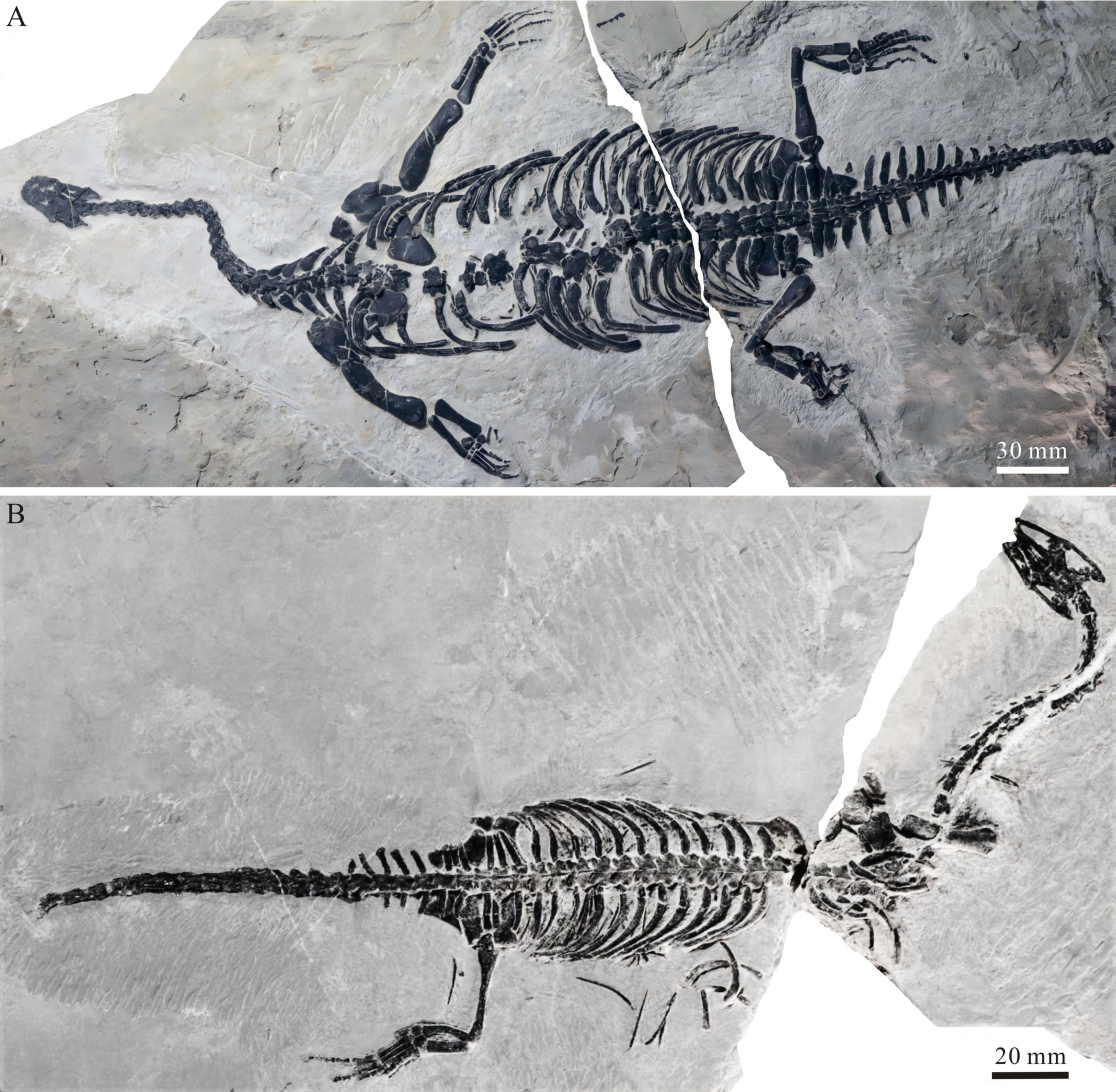
Two specimens of *Panzhousaurus rotundirostris*. (A) GMPKU-P-1059, holotype; (B) GMPKU-P-3241.

*Panzhousaurus rotundirostris* Jiang et al., 2019 is one of the few eosauropterygians from the early Middle Triassic (Anisian) Panxian Fauna in western Guizhou, China (Sun et al., 2006; Motani, 2009; Benton et al., 2014). Until recently, it was known only by two specimens preserved in the grey marly limestones from the Second (Upper) Member of the Guanling Formation in Xinmin, Panzhou, Guizhou (Fig. 1). *Panzhousaurus* was originally classified in the Eosauropterygia without reference to a particular clade (Jiang et al., 2019). Later analyses (Xu et al., 2022; Xu et al., 2023; Hu, Li & Liu, 2024) generally refer it to Pachypleurosauroidea or Pachypleurosauria (= Pachypleurosauridae of Lin et al., 2021; Liu et al., 2023) but competing hypotheses exist. Liu et al. (2025) recovered *Panzhousaurus* as a sister taxon to an unnamed clade comprising *Keichousaurus*, Simosauridae, *Brevicaudosaurus*, Nothosauridae plus Pistosauroidea within the Eusauropterygia, and Wang et al. (2025) recovered *Panzhousaurus* as a sister taxon to *Majiashanosaurus* within early branching eosauropterygians. The age of *P. rotundirostris* has been well constrained to the late Anisian (Pelsonian, 244.0 ± 1.3 Ma) by both biostratigraphic correlation and U-Pb dating (Sun et al., 2006; Wang et al., 2014). Along with *P. rotundirostris*, the coexisting vertebrates from the same biota include several other clades of marine reptiles and some actinopterygians (Li, 2003; Jiang et al., 2006a; Jiang et al., 2006b; Jiang et al., 2006c; Jiang et al., 2007; Jiang et al., 2008a; Jiang et al., 2008c; Li et al., 2006; Li et al., 2013; Xu & Shen, 2015; Ma, Xu & Geng, 2021).

**Figure 2:**
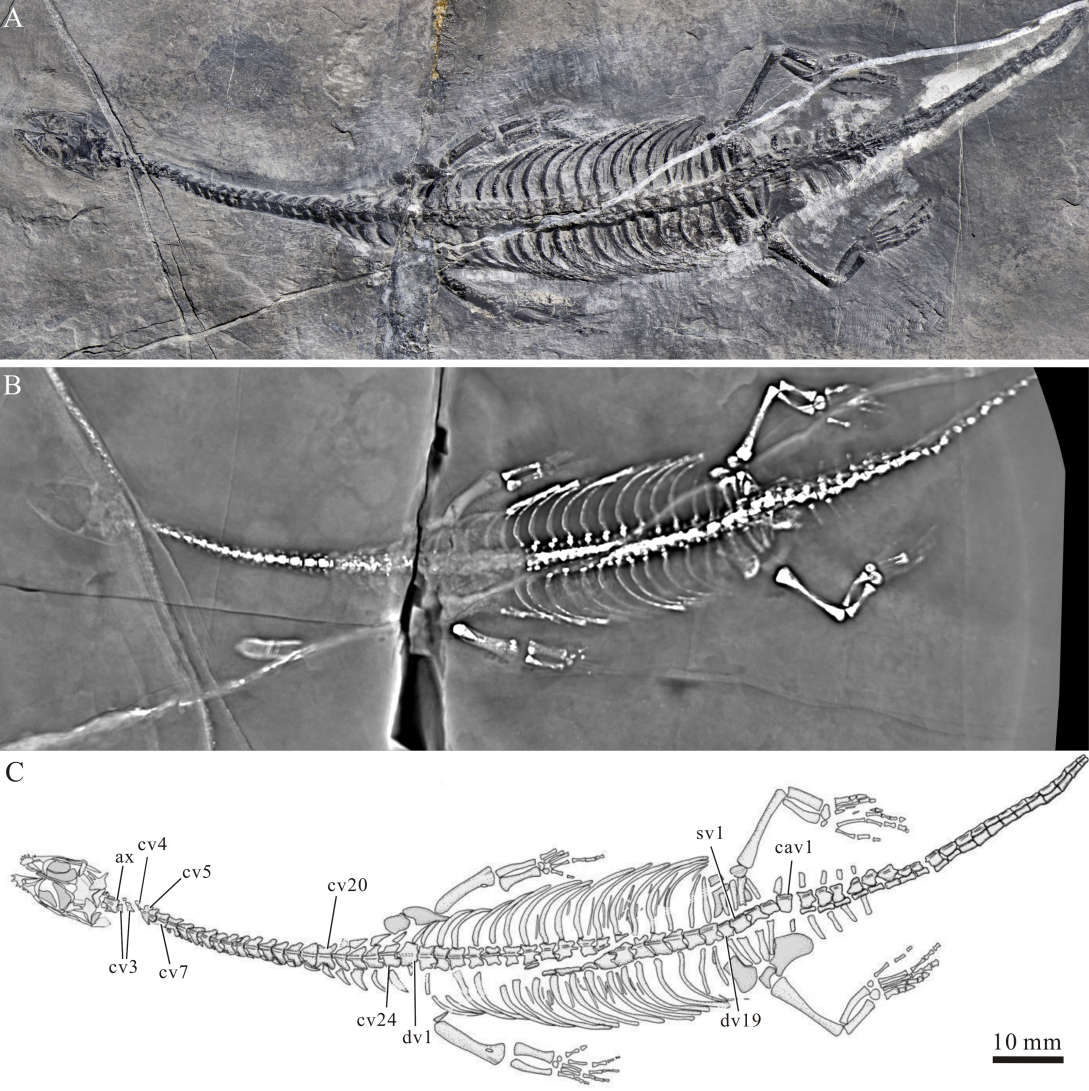
A nearly complete specimen of *Panzhousaurus rotundirostris*, IVPP V30747. (A) Photograph; (B) Computed laminography scanning slice; (C) Line-drawing.

Here, we describe the third specimen of *P. rotundirostris* (Fig. 2) from the same locality and horizon as the holotype. Comparative studies of this specimen with the type material reveal that some morphological details were previously undescribed or misidentified for this taxon, and consequently, a revision of *P. rotundirostris* is presented in this paper. In addition, we reassess the phylogenetic position of *Panzhousaurus* within Eosauropterygia using a cladistic approach. Hopefully, the present work contributes to a better understanding of the comparative anatomy, evolution and classification of early eosauropterygians in general.

## Material & Methods

The herein described specimen (IVPP V30747) of *Panzhousaurus rotundirostris* is curated at the fossil collections of the Institute of Vertebrate Paleontology and Paleoanthropology, Chinese Academy of Sciences (IVPP). The specimen was collected in 2008 and it was mechanically prepared by Meng-Nan Lü (IVPP) with sharp steel needles under an Olympus SZX7 microscope. Two previously described specimens of *P. rotundirostris* were reexamined, including GMPKU-P-1059 (holotype) and GMPKU-P-3241; both are curated at the Geological Museum of Peking University (GMPKU). The whole specimen and the pectoral girdle were scanned using the computed laminography scanner at the Institute of Vertebrate Paleontology and Paleoanthropology (IVPP), Chinese Academy of Sciences in Beijing, China, and the scan was set at the beam energy of 90 kV and flux of 50 µA. The former was with a resolution of 94.308 µm per pixel, and the latter was with a resolution of 24.807 µm per pixel (‘Figs. S1 and S2’ in https://doi.org/10.5281/zenodo.18167930).

### Phylogenetic analysis

A cladistic analysis was conducted to test the phylogenetic position of *Panzhousaurus* within Sauropterygia on the basis of a data matrix modified from Xu et al. (2023). The current data matrix includes 183 characters coded across 63 taxa; among them, eight character states of *Panzhousaurus* have been recoded according to this study (see ‘Supplementary Information 1’ in https://doi.org/10.5281/zenodo.18871621). All characters were unordered and equally weighted. We used the basal diapsid *Youngina capensis* for out-group comparison. Phylogenetic analysis was conducted in TNT 1.5 (Goloboff & Catalano, 2016) using a traditional heuristic search with 1,000 random addition replicates, saving up to 10 trees per replicate. Tree bisection-reconnection (TBR) branch swapping was performed under collapsing rule 3.

In addition, extended implied weighting (EIW; Goloboff, 2014; Goloboff, Torres & Arias, 2018; Ezcurra, 2024) analyses were conducted. The EIW settings, results, and a comparison with the equal-weight results are provided at Zenodo (https://doi.org/10.5281/zenodo.18871621, ‘Supplementary Information 2’).

**Data availability—**The raw data and computer code supporting this article are available at Zenodo: https://doi.org/10.5281/zenodo.18871621.

## Results

### Systematic paleontology

**Table:** 

Sauropterygia Owen, 1860
Eosauropterygia Rieppel, 1994
Pachypleurosauroidea Huene, 1956

**Diagnosis (emended from Liu et al., 2011; Lin et al., 2021)—**A superfamily distinguishable from other members of Eosauropterygia by the following combination of characters: presence of unretracted external nares (reversal in several pachypleurosaurids); presence of quadratojugal; presence of plate-like occiput with no distinct paroccipital process and with strongly reduced posttemporal fossae; exclusion of premaxilla from internal naris; absence of strongly projecting lateral ridge of surangular defining insertion area for superficial adductor muscle fibres on lateral surface of lower jaw; short mandibular symphysis; presence of trough on dorsal surface of retroarticular process; absence of distinct expansion of distal head of sacral rib; open ectepicondylar groove on humerus without anterior notch; and pubis with concave ventral (medial) margin.

**Content—** *Diandongosaurus* Shang, Wu & Li, 2011; *Majiashanosaurus* Jiang et al., 2014; *Dianmeisaurus* Shang & Li, 2015; Keichousauridae Young, 1965; and Pachypleurosauridae Nopcsa, 1928.

Keichousauridae Young, 1965

**Diagnosis (emended from Liu et al., 2011)—**A family distinguishable from other members of Pachypleurosauroidea by the following combination of characters: presence of T-shaped interclavicle; straight posterior margin of quadrate; straight anterior (preaxial) margin of radius; convex postaxial margin of ulna (reversal in *Panzhousaurus*); and five or more carpal ossifications.

**Content—** *Dawazisaurus* Cheng et al., 2016; *Dianopachysaurus* Liu et al., 2011; *Keichousaurus* Young, 1958; *Panzhousaurus* Jiang et al., 2019

*Panzhousaurus* Jiang et al., 2019

*Panzhousaurus rotundirostris* Jiang et al., 2019

**Holotype—**GMPKU-P-1059, an almost complete skeleton with some caudal vertebrae missing.

**Referred specimens—**GMPKU-P-3241 and IVPP V30747.

**Type locality and horizon—**Yangjuan Village, Xinmin Town, Panzhou City, Guizhou Province, China. Upper Member of Guanling Formation; Pelsonian, Anisian, Middle Triassic (Sun et al., 2006; Sun et al., 2014; Wang et al., 2014).

**Emended diagnosis—**A keichousaurid distinguishable from other members of this clade by the following autapomorphies: preorbital region short, 27.6–27.7% of skull length; nasal wedge-shaped, narrowing posteriorly and extending to level of anterior border of orbit; nasal process of premaxilla extending to level of anterior margin of prefrontal; 24 cervical and 19–20 dorsal vertebrae; phalangeal formula 3-4-5-4-3 for manus, and 2-3-4-5-3 for pes; and by the following exclusive combination of characters: paired frontals with very narrow interorbital portion; presence of six premaxillary teeth; cervical region slightly shorter than trunk region.

### Description and comparison

The new specimen (IVPP V30747) is nearly complete and fully articulated in dorsal view, only lacking part of the pectoral girdle and a distal portion of the tail (Fig. 2). It is notably smaller than the holotype (Table 1) and measures 171.1 mm from the premaxillary symphysis to the last preserved (21st) caudal vertebra. The snout–vent and standard (last four dorsal vertebrae) lengths are 124.6 mm and 11.4 mm, respectively, *versus* 274.9 mm and 22.0 mm in the holotype. The small size and absence of any ossified distal carpals and tarsals indicate that the new specimen represents a juvenile.

**Table 1:** Measurements of *Panzhousaurus rotundirostris* (in mm).

	GMPKU-P-1059	GMPKU-P-3241	IVPP V30747
Snout length	5.1	?	3.9
Skull length	18.4	∼17.5	14.4
Snout-Vent length	274.9	135.0	124.6
Standard length (last four dorsal vertebrae)	22.0	13.2	11.4
Length of external naris	?	?	?
Length of orbit	5.4	?	5.2
Length of axis to last cervical vertebra	93.0	?	48.2
Length of axis to last dorsal vertebra	233.0	114.7	101.1
Length of mandible	23.6	?	16.9
Length of humerus	29.0	15.8	11.7
Proximal width of humerus	6.2	3.1	1.8
Distal width of humerus	7.6	4.9	3.6
Length of radius	15.0	?	6.0 (R)
Length of ulna	15.0	?	6.3 (R)
Length of metacarpal I	3.8	?	2.0
II	5.6	?	3.7
III	7.0	?	4.3
IV	4.9	?	4.2
V	3.9	?	2.6
Length of femur	28.0	15.5 (R)	13.0
Proximal width of femur	4.4	3.7 (R)	2.9
Distal width of femur	3.2	2.1 (R)	1.9
Length of fibula	14.0	7.1 (R)	6.2
Length of tibia	13.0	6.9 (R)	5.5
Length of metatarsal I	4.7	2.7	2.5
II	8.4	4.9	4.6
III	9.0	5.8	5.3
IV	8.8	5.8	4.9
V	6.9	4.3	3.4

**Notes.**

R right

**Skull—**The skull is 14.4 mm from the premaxillary symphysis to the occipital condyle, and the snout (preorbital) region is very short, 3.9 mm in length, accounting for 27.1% of the skull length in the new specimen. This ratio is comparable to that (27.7%) in the holotype. The snout is round anteriorly and lacks a rostral constriction. The external naris is relatively small compared with that of *Keichousaurus*, and its posterior margin is mainly formed by the anterior margin of the nasal, similar to the condition in *Dianopachysaurus* (Liu et al., 2011)*.* The orbit is large and oval, about three times as long as the supratemporal fenestra in the new specimen. In the holotype, however, the orbit is relatively smaller, about 2.5 times as long as the supratemporal fenestra.

**Figure 3:**
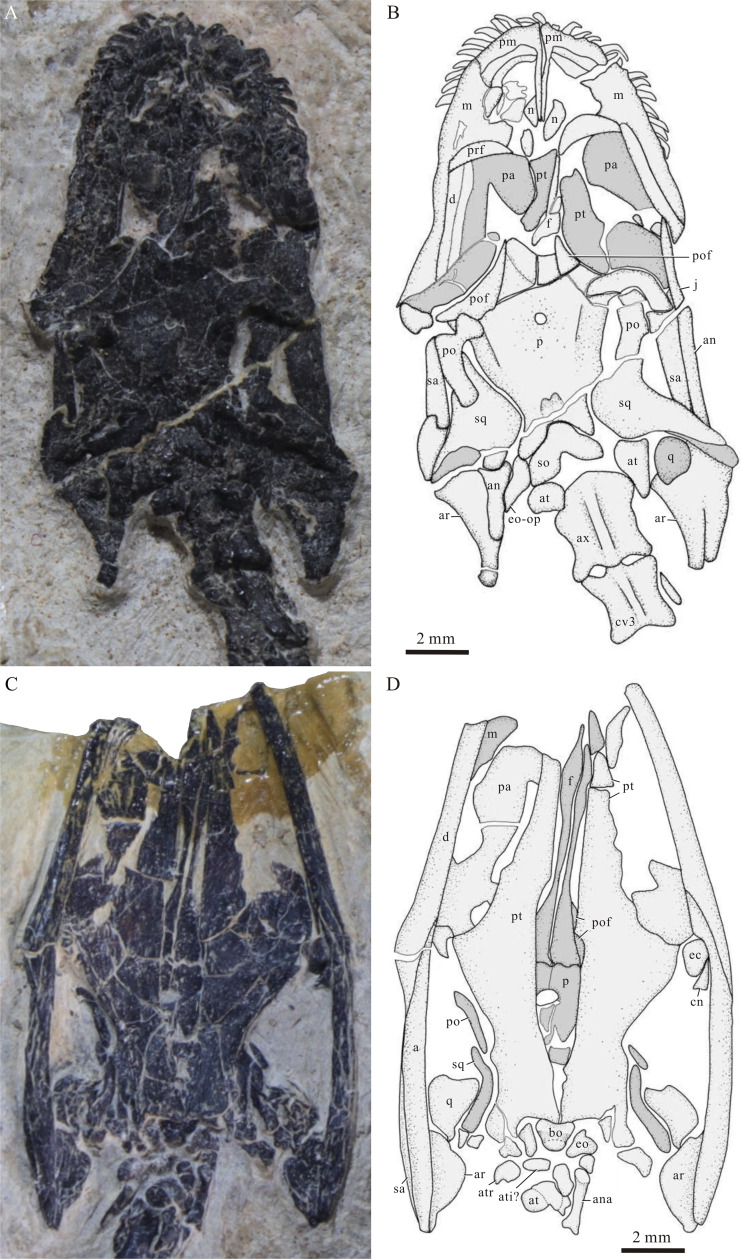
Skulls of *Panzhousaurus rotundirostris*. (A) Photograph of GMPKU-P-1059; (B) line-drawing of GMPKU-P-1059; (C) photograph of GMPKU-P-3241; (D) Line-drawing of GMPKU-P-3241.

**Figure 4:**
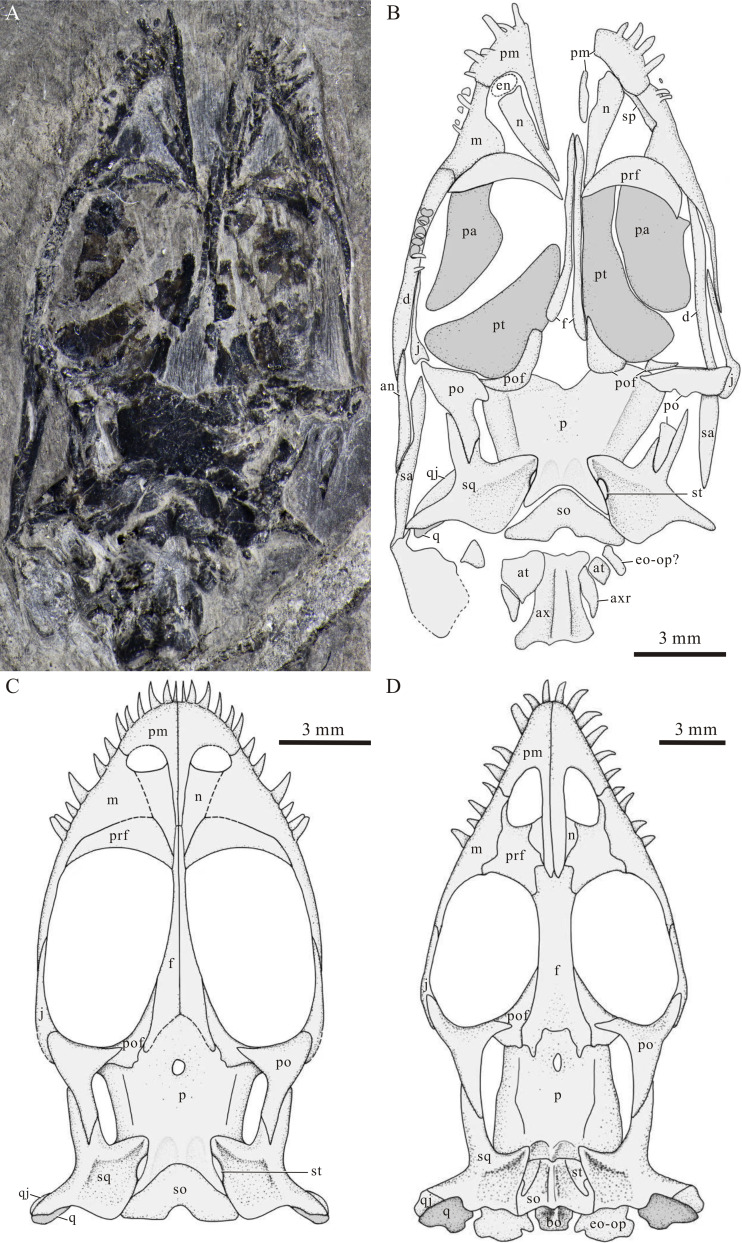
Skull of *Panzhousaurus rotundirostris* and comparison with that of *Keichousaurus hui*. (A) Photograph of IVPP V30747; (B) line-drawing of IVPP V30747; (C) reconstruction of the skull of *P. rotundirostris*; (D) reconstruction of the skull of *K. hui* (modified from Xu et al., 2025).

The paired premaxillae are inverted L-shaped, forming most of the preorbital region (Figs. 3 and 4). Each premaxilla occupies about three-fourths of the preorbital length. It has a long nasal process that extends posteriorly to the level of the anterior margin of the prefrontal and contacts the anterior tip of the frontal. The posterolateral process of the premaxilla is relatively short and contacts the maxilla posteriorly at the level of the center of the external naris. Because of poor state of preservation, the sutures of the premaxillae with surrounding bones are hard to discern in the holotype. Jiang et al. (2019) illustrated a strong and posteriorly expanded ‘posterolateral process’ and a rather short nasal process for the premaxilla but our re-examination shows that the former actually includes part of the mandible, and the latter is longer than that of the previous interpretation (Fig. 3B).

The nasals are wedge-shaped, distinctly longer in ratio than those previously described in the holotype (Jiang et al., 2019). The anterior margin of the nasal forms the posterior border of the external naris. Posteriorly, the nasal tapers into a pointed tip and inserts between the prefrontal and the frontal (Fig. 4B). The left nasal is separated from contact with the right one by the nasal processes of the premaxillae and the anterior tips of the frontals. The nasals are much poorly preserved and hard to identify in the holotype, and they are almost missing in GMPKU-P-3241.

The maxilla is elongate, bearing a triangular ascending process that contacts the nasal anteromedially and the prefrontal posteromedially. The anterior process of the maxilla is short and contributes to the lateral margin of the external naris (Fig. 4B). Posteriorly, the maxilla bears a long posterior process that contacts the anterior process of the jugal and both form the lateral margin of the orbit (Fig. 4B). Notably, the maxilla is poorly preserved in the holotype. Jiang et al. (2019) illustrated a very strong ‘maxilla’ with a massive ascending process for *Panzhousaurus*. However, our re-examination of the holotype reveals that the previously illustrated ‘maxilla’ would include part of the prefrontal and mandibular bones besides the real maxilla (Fig. 3B).

The jugals are better preserved in the new specimen (Fig. 4B) than those in the holotype (Fig. 3B). Each jugal has a long and slender anterior process that anteriorly contacts the posterior process of the maxilla and ends at the level of the orbital center. Posteriorly, the jugal curves slightly dorsally and contacts the descending process of the postorbital medially. The posterior process of the jugal appears short and does not contact the anterior process of the squamosal. Our re-examination reveals that the ‘plate-like jugals’ previously identified in the holotype (Jiang et al., 2019) would include part of the mandible.

The prefrontal is well-preserved in the new specimen (Fig. 4B), notably larger than the previous interpretation from the holotype (Jiang et al., 2019). The prefrontal is crescent-shaped, tapering at both ends. The anterior margin of the prefrontal is convex and contacts the maxilla laterally and the nasal and frontal medially, and the posterior margin of the bone is concave and defines the anterior margin of the orbit. The prefrontals are hard to discern in the holotype because of poor state of preservation. A rather ‘small prefrontal’ at the anteromedial corner of the orbit was previously described in the holotype (Jiang et al., 2019), but our re-examination reveals that this could be only a small medial part of the real bone.

The paired frontals are incompletely preserved in the new specimen and the holotype but better preserved in ventral view in GMPKU-P-3241 (Figs. 3C, 3D, 4B). Each frontal has an elongate main body, and its lateral margin is slightly concave, forming most of the medial border of the orbit. The interorbital septum is very narrow, similar to the condition in *Dianmeisaurus* (Hu, Li & Liu, 2024). Anteriorly, the frontal tapers to a pointed tip and extends slightly beyond the posterior end of the dorsal tip of the premaxilla (Fig. 3D). Posteriorly, the frontal slightly expands and contacts the postfrontal laterally and the parietal posteriorly. The frontals are poorly preserved in the holotype; our reexamination shows that the ‘relatively short and broad frontals’ previously illustrated by Jiang et al. (2019) could include part of the pterygoid (Fig. 3B).

The left postfrontal is nearly complete in the new specimen. It is nearly triangular and forms the posteromedial border of the orbit (Fig. 4B). The anterior process of the postfrontal contacts the posterolateral process of the frontal and its ventral (lateral) process contacts the dorsal process of the postorbital. Jiang et al. (2019) identified a ‘long posterior process’ in the postfrontal that forms the medial margin of the supratemporal fenestra in the holotype, but our re-examination shows that this process actually belongs to the lateral part of the parietal (Fig. 3B).

The postorbital is triradiate. The descending (lateral) process contacts the posterior end of the jugal and forms the major part of the postorbital bar between the orbit and the infratemporal fenestra (Fig. 4B). The posterior process of the postorbital is longer than that previously interpreted in the holotype (Jiang et al., 2019). It inserts into the forked anterior process of the squamosal posteriorly and both together form the bar between the supratemporal fossa and the ventrally open infratemporal fenestra (Fig. 4B).

The squamosal is better preserved in the new specimen than that in the holotype (Figs. 3B, 4B). It is large and irregular, forming the posterior half of the supratemporal arch. The squamosal bears a long anterolateral process and a short medial process on the skull roof portion. The anterolateral process is forked, receiving the pointed tip of the posterior process of the postorbital. The medial process extends medially and inserts into the posterior portion of the parietal. The descending process of the squamosal extends ventrolaterally, contacts the quadratojugal anteriorly, and reaches the quadrate condyle. Posteriorly, the squamosal has a posteromedial flange at the occipital portion, which medially contacts the posterolateral process of the parietal, the supratemporal, and the supraoccipital (Fig. 4B).

The left quadratojugal is discernible in the new specimen (Fig. 4B), contacting the descending process of the squamosal posteriorly. It is small and narrow, tapering dorsally. The quadrate is well exposed in GMPKU-P-3241 (Fig. 3D); it is roughly triangular with a ventral condyle contacting the articular.

The parietals are fused into a broad median ossification (Figs. 3B, 4B). The median pineal foramen is anteriorly located at the parietal (Figs. 3B, 3C), similar to the conditions in other keichousaurids (Lin & Rieppel, 1998; Liu et al., 2011). The region just anterior to the pineal foramen was broken away in the new specimen (Fig. 4B). The middle portion of the parietal is slightly constricted for the supratemporal fenestra. The parietal has a laterally descending flange along the medial margin of the supratemporal fenestra on each lateral side of the bone (Figs. 3B, 4B), which would serve as the area of attachment of the jaw adductor musculature as in *Youngina* and many other diapsids (Carroll, 1981). Posteriorly, the parietal has a slightly concave occipital edge, and just posterior to the edge, the bone slopes down and bears a pair of relatively deep posteroventral processes. The posteromedial margin of this process contacts the supraoccipital.

The new specimen shows that the posteroventral process of the parietal laterally contacts the posteromedial flange of the squamosal and a small and narrow bone on each side of the skull (Fig. 4B). This bone is similar to the supratemporal of *Keichousaurus* in morphology and position (Figs. 4C, 4D; Lin & Rieppel, 1998; Holmes, Cheng & Wu, 2008; Xu et al., 2025). Following the terminology, we identify this bone as the supratemporal here for *Panzhousaurus.* Because of its small size and poor state of preservation, the supratemporal is hard to identify in the holotype. The supratemporal is rarely known in other pachypleurosauroids but is a plesiomorphic character in neodiapsids (*e.g.*, *Youngina* and *Claudiosaurus*), Ichthyosauromorpha, Thalattosauria and Saurosphargidae (Li et al., 2013).

The supraoccipital is relatively broad and roughly triangular, forming the posterior roof of the braincase. The sutures between the supraoccipital and its neighbor bones (*e.g*., parietal and squamosals) are hard to discern in the holotype, and Jiang et al. (2019) failed to identify the supraoccipital and described an extremely ‘long parietal’ (even longer than the frontal) for *Panzhousaurus*. However, our reexamination and comparison with the new specimen shows that this ‘parietal’ actually includes the whole supraoccipital and the posterior processes of the frontals (Fig. 3B).

The palate is mostly unknown in the dorsally exposed holotype and new specimen described here but it is relatively well known from GMPKU-P-3241 (Fig. 3D). The latter has been described in detail by Lin et al. (2021), whose descriptions are largely agreed with our reexamination. The long and roughly trapezoid pterygoids and the relatively small and plate-like palates are the main components of the palate. Additional components include a pair of small trapezoid ectopterygoids. Ectopterygoids are also present in *Diandongosaurus, Dianmeisaurus* and *Keichousaurus* (Shang, Wu & Li, 2011; Shang, Wu & Li, 2017; Xu et al., 2025), but they are unknown or absent in pachypleurosaurids.

**Mandible—**The dentary is more or less discernable from all three available specimens. It is elongate and extends posteriorly to the level of the middle portion of the parietal, where it contacts the surangular and angular posteriorly (Figs. 3, 4B). The surangular, well preserved in the new specimen, is also elongate and forms at the posterodorsal portion of the mandible (Fig. 4B).

The angular is well exposed ventrally in GMPKU-P-3241 (Fig. 3D). It is wedge-shaped, contacting the dentary anteriorly and the surangular dorsally. In GMPKU-P-3241, the angular almost extends posteriorly to the level of the posterior of the articular, accounting for approximately 50% of the estimated total mandibular length. This condition differs from that in *Keichousaurus* (Xu et al., 2025), where the angular is only 18.7% of mandibular length and extends posteriorly to connect with the articular and prearticular. Additionally, in GMPKU-P-3241, the angular directly overlaps the articular and prearticular ventrally, whereas in *Diandongosaurus*, the angular connects with these bones posteromedially (Sato et al., 2014).

The splenial is partly seen in the holotype and new specimen, forming the lingual surface of the anterior portion of the mandible (Figs. 3 and 4B). Posteriorly, the articular and prearticular can be seen in the holotype and GMPKU-P-3241, forming a moderately developed retroarticular process (Fig. 3).

**Dentition—** Five teeth are discernible along the oral margin of the left premaxilla in the new specimen. Considering an obvious gap for a missing tooth, the complete number would be six, consistent with the count in the holotype. The morphology of the premaxillary tooth is more complete known from the holotype (Fig. 3B). They are homodont, having a pointed and procumbent tip, similar to those in *Keichousaurus* (Liao et al., 2021; Xu et al., 2025). The maxillary and dentary teeth are partly discernible from the new specimen and the holotype, and they are similar to the premaxillary teeth in shape but are slightly smaller in size. However, their numbers are uncertain because of occlusion of jaws.

**Axial skeleton—**There are 24 cervical vertebrae (including the atlas-axis complex) in the new specimen, consistent with the count in the holotype. The atlas is represented by a pair of trapezoid neural arches behind the supraoccipital. The axis centrum is somewhat longer than the third cervical. Posterior to the axis, the cervical vertebrae are discernible in GMPKU-P-3241 in ventral view (Fig. 1B) and they are notably longer than wide. The cervicals show pachyostotic neural arches with a low neural spine (Figs. 1 and 2). The associated ribs increase gradually in size toward the trunk vertebrae posteriorly.

**Figure 5:**
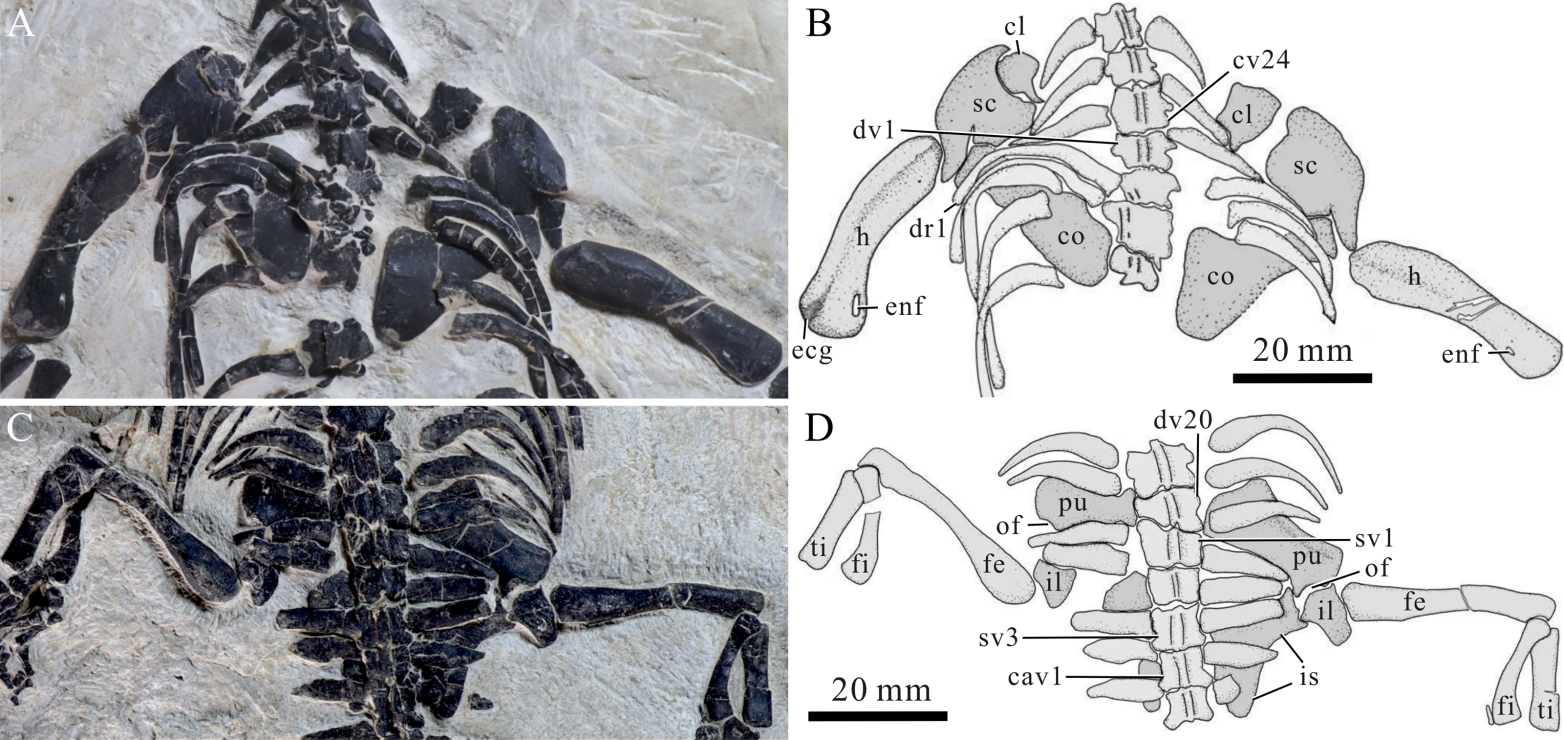
Pectoral and pelvic girdle of *Panzhousaurus rotundirostris*, GMPKU-P-1059. (A) Pectoral girdle, photograph; (B) pectoral girdle, line-drawing; (C) pelvic girdle, photograph; (D) pelvic girdle, line-drawing.

**Figure 6:**
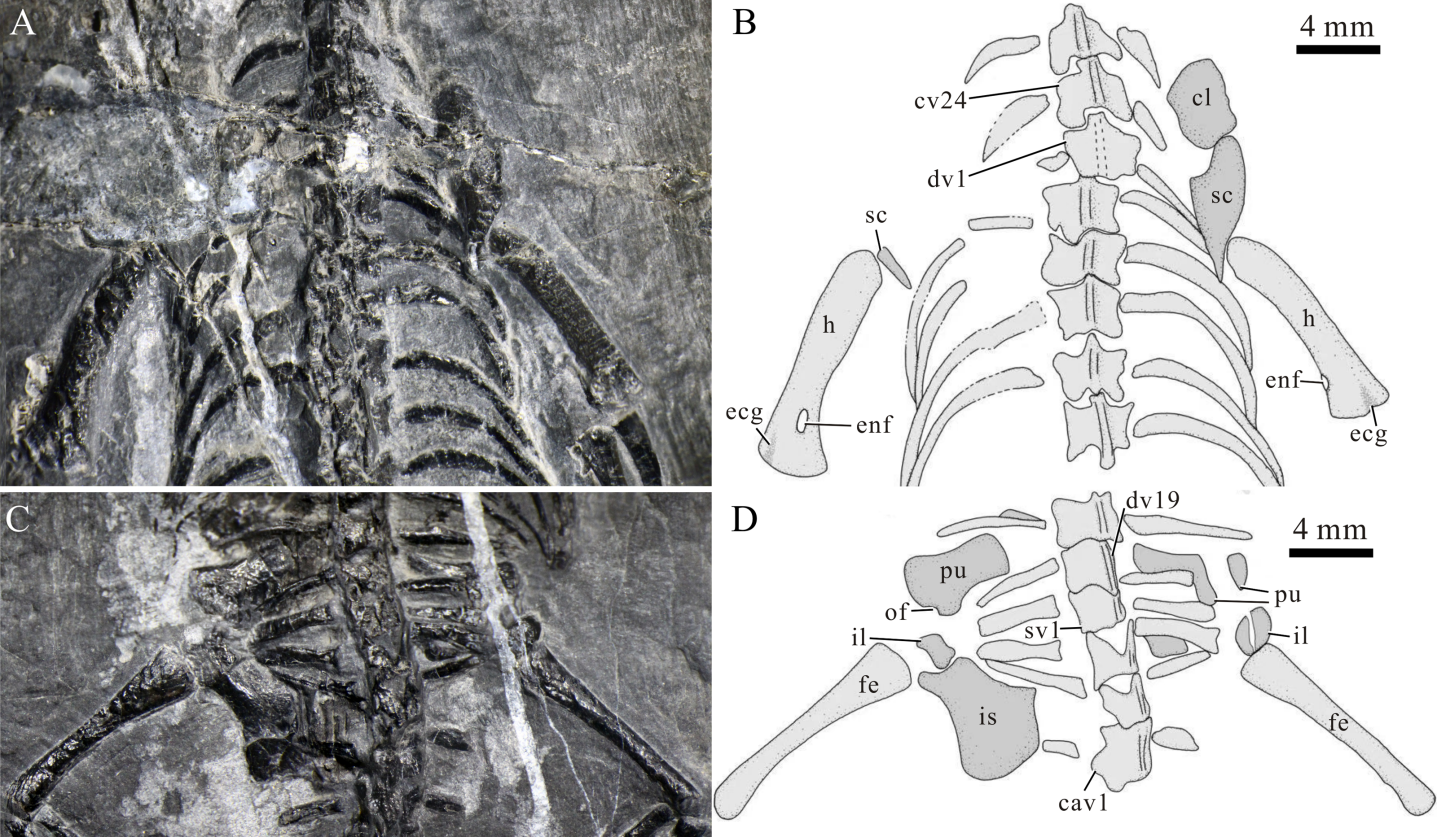
Pectoral and pelvic girdle of *Panzhousaurus rotundirostris,* IVPP V30747. (A) Pectoral girdle, photograph; (B) pectoral girdle, line-drawing; (C) pelvic girdle, photograph; (D) pelvic girdle, line-drawing.

The dorsal vertebrae are exposed in dorsal view in all three available specimens (Figs. 1 and 2). There are 19 dorsal vertebrae in the new specimen, slightly less than the count (20) in the holotype. The 25th vertebra is considered the first dorsal vertebra because its rib is notably longer than the last cervical rib (Fig. 2). The transverse processes of the dorsal vertebrae are stout, more prominent than those of the cervical series. The zygapophyses are broad and flat with nearly horizontal articular surfaces, and the neural spines are low. The dorsal ribs are single-headed and pachyostotic proximally. Most ribs are long and bow posterolaterally (Figs. 1 and 2), with the exception of the last dorsal rib, which is short and only slightly curved. Additionally, three sacral vertebrae are present. The sacral ribs are slightly shorter than the last dorsal rib and almost straight, converging toward the ilium (Figs. 5D, 6D). Some elements of the gastralia are discernible through the interval of dorsal ribs; they are long and slender, rod-like bones.

The caudal vertebrae are incompletely preserved with 21 vertebrae discernible in the new specimen (15 in the holotype and 22 in GMPKU-P-3241). Within the Keichousauridae, *Dawazisaurus* and *Keichousaurus* are known about their complete series of caudal vertebrae with 37 vertebrae in the former and ∼40 in the latter (Wen et al., 2022). Following their counts, we tentatively reconstruct 37 vertebrae for *Panzhousaurus.* In the holotype, 11 pairs of caudal ribs are present, representing the most complete count in *Panzhousaurus.* The neural spines of caudal vertebrae are low, as in other keichousaurids. Five and eight pairs of caudal ribs are known in the new specimen and GMPKU-P-3241, respectively. The anterior caudal ribs also show pachyostosis; the first caudal rib is the longest but slightly shorter than the last sacral rib, and other ribs gradually reduce in length posteriorly.

**Appendicular skeleton—**In the pectoral girdle, the clavicles, scapulae and coracoids are exposed in dorsomedial view and the interclavicle is not exposed (Figs. 5 and 6). The clavicle is largely obscured by covering ribs and vertebrae. It is attached to the medial surface of the scapula and has an anterolaterally expanded corner. However, it lacks an anterolateral process found in *Diandongosaurus* and *Dianmeisaurus* (Shang, Wu & Li, 2011; Shang, Wu & Li, 2017). The scapula consists of a broad, proximal (ventral) portion and a triangular distal (dorsal) blade (Figs. 5B, 6B). This dorsal process becomes thin and tapers to a point distally (Fig. 6B), similar to the conditions in *Dawazisaurus* (Cheng et al., 2016) but unlike the blunt tips of *Honghesaurus* and *Luopingosaurus* (Xu et al., 2022; Xu et al., 2023). A right coracoid is well exposed in the holotype; it is largely trapezoidal with the distal portion more expanded than the proximal portion. The anterior margin is distinctly concave and the posterior margin is only slightly concave (Fig. 5B).

The humerus is bowed posteromedially with a slightly expanded distal end, a convex anterolateral margin and a concave posteromedial margin (Figs. 5 and 6). The ectepicondylar groove is discernible near the anterolateral margin of the distal end of the bone (Fig. 5B). The entepicondylar foraminen is clearly discernible and the deltopectoral crest is only weakly developed (Figs. 5B, 6B). The radius is approximately as long as the ulna; the former is slightly expanded proximally, having a straight or slightly convex anterior (preaxial) margin and a slightly concave posterior margin. The ulna, stronger than the radius, has a straight shaft, and the proximal and distal ends of the bone are similarly expanded.

**Figure 7:**
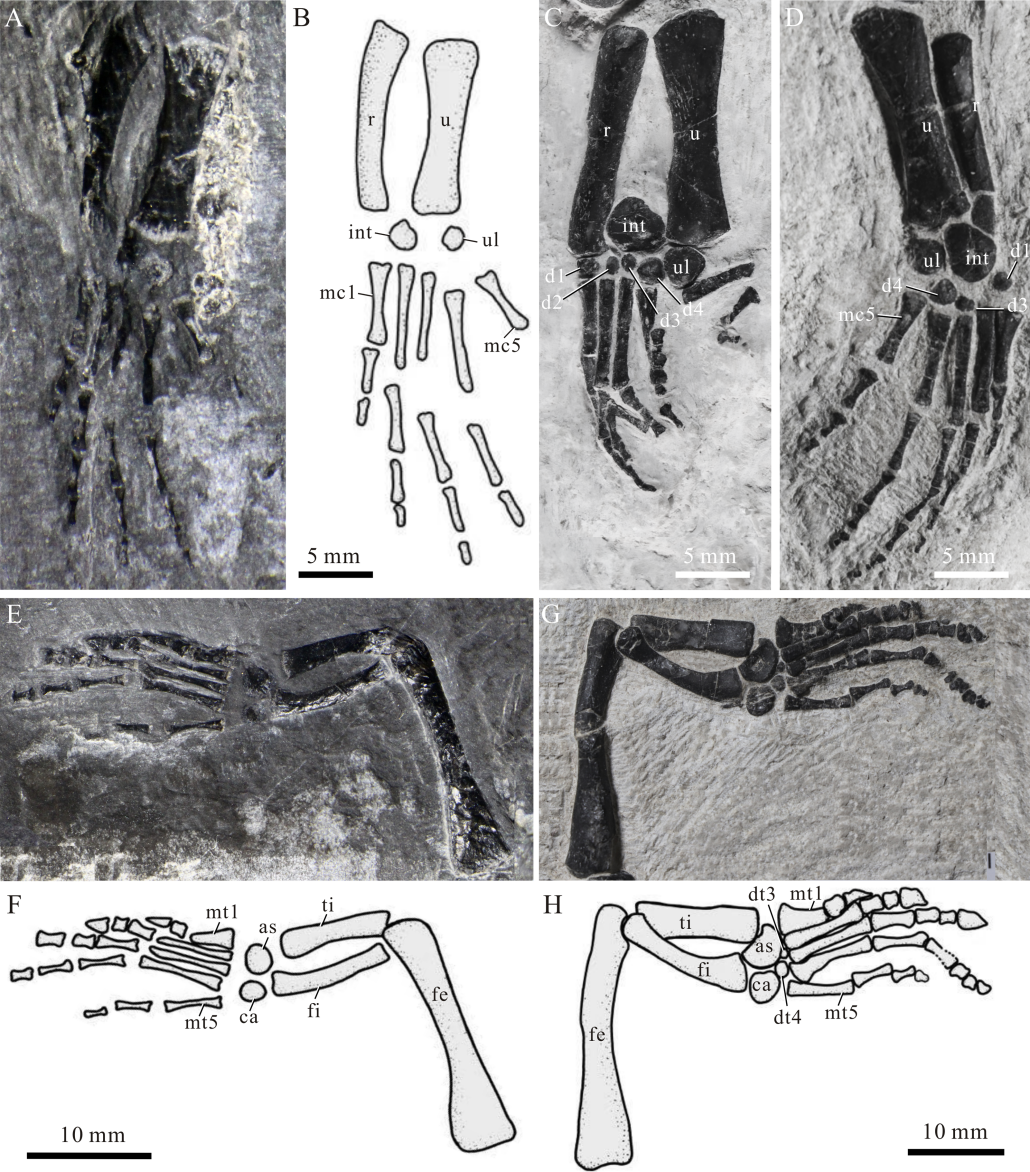
Limbs of *Panzhousaurus rotundirostris*. (A) Left forelimb of IVPP V30747, photograph; (B) left forelimb of IVPP V30747, line-drawing; (C) left forelimb of GMPKU-P-1059, photograph; (D) right forelimb of GMPKU-P-1059, photograph; (E) left hind limb of IVPP V30747, photograph; (F) left hind limb of IVPP V30747, line-drawing; (G) right hind limb of GMPKU-P-1059, photograph; (H) right hind limb of GMPKU-P-1059, line-drawing.

There are six ossified carpals (intermedium, ulnare, and distal carpals I–IV) in the left manus of the large, adult holotype, whereas in the juvenile new specimen, only the intermedium and ulnare are present and the distal carpals are not ossified. The ulnare and intermedium are subcircular; the former is smaller than the latter (Figs. 7B, 7D). The ulnare lies close to the distal end of the ulna, and the intermedium is positioned between the distal ends of the radius and ulna. Four tiny, round distal carpals are close to the proximal ends of metacarpals I–IV, respectively. Five metacarpals are best preserved in the holotype (Figs. 7C, 7D), and they are straight with slightly expanded proximal and distal ends. The first is the shortest and the third is the longest. The phalangeal count is 3–4–5–4–3, indicating a slight hyperphalangy in the manus (Fig. 7D) of *Panzhousaurus* (Figs. 7C, 7D).

The pelvic girdle is partly exposed in dorsal view, comprising a pair of pubes, ilia and ischia (Figs. 5D, 6D). The pubis is better exposed in the holotype. It is broad and hourglass-shaped, constricted in its middle portion. The obturator foramen lies in the posterior margin of the pubis close to the sutures with the ischium and ilium. The ilium is small and stout, having a ventrally expanded acetabular portion and a short dorsal blade. The ischium is a large, plate-like bone with its proximal (dorsal) portion contacting the ventral part of the ilium and the posterodorsal part of the pubis. The middle portion of the ischium is also constricted and the distal portion is more expanded than the proximal portion.

The hindlimbs are well-preserved in the holotype and the new specimen (Figs. 7E, 7G). The femur is nearly as long as the humerus but more slender than the latter. It has a straight shaft with a constricted middle portion; the proximal end is more expanded than the distal end. The tibia is robuster and shorter than the fibula. It has a straight shaft with slightly expanded proximal and distal ends. The fibula has a curved shaft with a strongly concave medial margin and a slightly convex lateral margin.

In the pes, the calcaneum and astragalus are consistently ossified in all three specimens; the former is small and rounded, and the latter is larger and kidney-shaped (Figs. 7F, 7H). Two distal tarsals (III and IV) are present in the holotype and a single tarsal (IV) in GMPKU-P-3241. However, the distal tarsals are absent in the juvenile new specimen. Five metatarsals are well preserved. Metatarsal I is the shortest and stoutest phalange with an expanded proximal end, and Metatarsal III is the longest. The phalangeal formula is 2–3–4–5–4. The unguals of the second and third digits are distinctly expanded (Fig. 7H).

### Phylogenetic analysis

**Figure 8:**
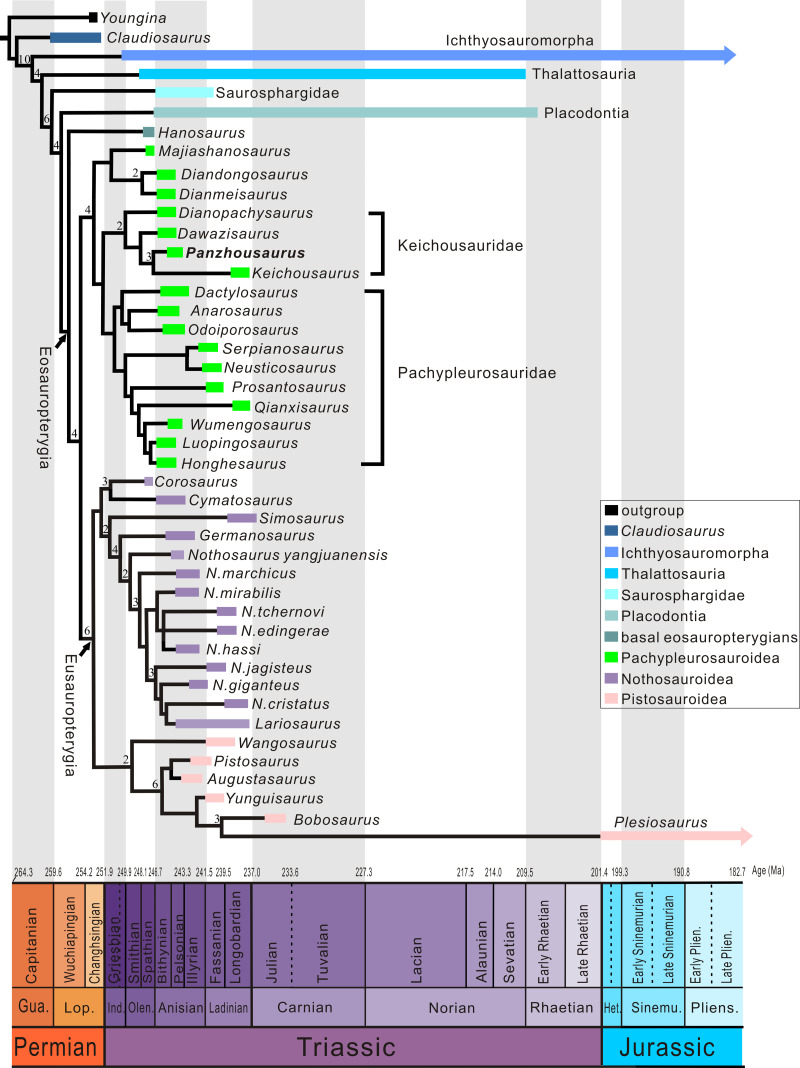
Phylogenetic position of *Panzhousaurus rotundirostris*. Strict consensus of two trees rooted with *Youngina* (TL = 864, CI = 0.269, RI = 0.684). Bremer decay indices larger than 1 are indicated above the nodes of the tree.

Phylogenetic analysis recovered two most parsimonious trees (tree length = 864 steps, consistency index = 0.269, retention index = 0.684). As shown in the strict consensus tree (Fig. 8; Fig S4 in https://doi.org/10.5281/zenodo.18167930), *Panzhousaurus* is recovered as a sister taxon to *Keichousaurus*, well supported (Bremer Index = 3) by the following synapomorphies: separation of nasals by elongated premaxillary processes posteriorly contacting frontals, absence of a distinct L-shaped jugal, presence of a supratemporal bone, and presence of a distinct but low coronoid process.

*Panzhousaurus*, together with *Dawazisaurus*, *Dianopachysaurus* and *Keichousaurus* are united within the clade Keichousauridae, supported (Bremer Index = 2) by the following characters: presence of a quadrate with a straight posterior margin; radius with straight anterior (preaxial) margin; ulna with a convex posterior margin; and five or more carpal ossifications. Additionally, presence of a T-shaped interclavicle has been recognized as a keichousaurid synapomorphy but this feature is unknown in *Panzhousaurus* because the interclavicle is not exposed in all available specimens.

*Majiashanosaurus*, *Diandongosaurus* and *Dianmeisaurus* are united as an unnamed clade at the base of Pachypleurosauroidea. The monophyly of this clade is supported by the following synapomorphies: presence of unretracted external nares (reversal in several pachypleurosaurids); presence of a plate-like occiput with no distinct paroccipital process and with strongly reduced posttemporal fossae; presence of a trough on the dorsal surface of the retroarticular process; absence of a distinct expansion on the distal head of the sacral rib; presence of an open ectepicondylar groove on the humerus without an anterior notch; and presence of a pubis with a concave ventral (medial) margin.

The sister-group relationships between Pachypleurosauroidea and Eusauropterygia are well supported (Bremer Index = 4) by a series of characters: presence of an exposed quadrate in lateral view (independently evolved in *Chaohusaurus* and thalattosaurs); presence of a CP ratio (ratio between the numbers of cervical vertebrae to all presacral vertebrae) of 0.50 or more (reversal in pachypleurosaurids and most nothosauroids); clavicle with an anterolaterally expanded corner; coracoid with a strongly waisted middle portion; presence of a thyroid fenestra; and presence of a hyperphalangy in manus (reversal in some pachypleurosaurids and a few nothosauroids). This is largely consistent with the previous scenarios of some authors (*e.g.*, Rieppel & Lin, 1995; Liu et al., 2011; Neenan, Klein & Scheyer, 2013; Lin et al., 2021; Xu et al., 2022; Xu et al., 2023; but see Wang et al., 2022; Wang et al., 2025; Liu et al., 2025). Notably, Rieppel (1998), Rieppel (1999) and Rieppel (2000) proposed a paraphyletic Eusauropterygia but later Rieppel (2002) restored the monophyly of this clade.

Additionally, *Hanosaurus* is recovered at the base of Eosauropterygia. This taxonomic assignment, although weakly supported (Bremer Index = 1), agrees with some of the previous scenarios (Jiang et al., 2019; Liu et al., 2025). By contrast, Lin et al. (2021) recovered *Hanosaurus* at the base of Pachypleurosauroidea, and Wang et al. (2022) and Wang et al. (2025) removed it outside of Eosauropterygia. We notice that the interrelationships of Eosauropterygia remain highly unstable, and even the monophyly of the Pachypleurosauroidea and Eusauropterygia was challenged by some analyses (*e.g.*, Wang et al., 2025). Nevertheless, a further comprehensive analysis of eosauropterygian relationships is out of scope of this paper.

## Discussion

### IVPP V30747 as a juvenile of *Panzhousaurus rotundirostris*

A comparison of the juvenile specimen (IVPP V30747) with the holotype and referred specimen confirms its attribution to *P. rotundirostris* and documents its ontogenetic features.

IVPP V30747, like the holotype and GMPKU-P-3241, was excavated from the Second (Upper) Member of the Guanling Formation of Yangjuan, Xinmin, Panzhou, Guizhou. The new specimen is nearly consistent with the holotype and GMPKU-P-3241 in osteology, *i.e.*, the similar ratio (27.1–27.7%) of preorbital region to skull length, presence of anteriorly located and unretracted external nares, the nasal process of the premaxilla extending to the level of the anterior margin of prefrontal, paired frontals with a very narrow interorbital portion, fusion of parietals into a broad median ossification, six teeth in each premaxilla, almost the same count of vertebrae (24 cervical and 19-20 dorsal vertebrae) and similar appendicular skeleton (see Description above). Some minor differences (see below) do exist between IVPP V30747 and the holotype, but these most likely reflect different ontogenetic stages (juvenile *vs.* adult) of *P. rotundirostris*.

IVPP V30747 is considered here as a juvenile based on features that differ from the holotype and referred specimen. First, it has a relatively larger orbit; the ratio of orbit length to skull length is 0.36 in the new specimen, contrasting 0.29 in the holotype. As shown by Griffin et al.’s (2021) study of reptile ontogeny, a relatively large orbit often indicates immaturity. Second, it has fewer (six pairs) caudal ribs than the holotype (11 pairs). A gradual increase in caudal rib number is known from the growth of the pachypleurosaurid *Neusticosaurus* (Sander, 1989), and the discovery of this new specimen provides evidence that this increase also occurs in keichousaurid growth.

Additional features support the juvenile status of IVPP V30747. It has a notably smaller body size (124.6 mm in snout–vent length) than that of the holotype (274.9 mm in snout–vent length). It has clear sutures in the skull, and the neural arches associated with some dorsal vertebrae are not fused. Similar unfused neural arches are also present in the juveniles of other types of sauropterygians (Klein & Scheyer, 2014; Liu et al., 2025). It preserves only the proximal carpal/tarsal pair and lacks any ossified distal carpals and tarsals, a feature indicating immaturity in other pachypleurosauroids (Sander, 1989; Lin & Rieppel, 1998). In contrast, the slightly larger GMPKU-P-3241 (135.0 mm in snout–vent length) with a single ossified distal tarsal likely represents an intermediate ontogenetic stage between the juvenile new specimen and the adult holotype (with three ossified distal tarsals).

### Implications for the keichousaurid origin

The genus *Keichousaurus* was originally classified by Young (1958) in the Pachypleurosauridae but later in its own family Keichousauridae (Young, 1965). For a long time, *Keichousaurus* was the only taxon of this family, and its origin and paleobiographic evolution were obscure. Storrs (1991) first performed a cladistic analysis illuminating the relationships of *Keichousaurus* with other sauropterygians, and he recognized a monophyletic Pachypleurosauridae with *Keichousaurus* nested within the Germanic pachypleurosaurids. This scenario would indicate a western Tethyan origin for *Keichousaurus* but it is not supported by later analyses. Rieppel & Lin (1995) made a review of pachypleurosaurs, and their analysis recovered *Keichousaurus* as the sister taxon to all other pachypleurosaurs. Accordingly, they support Young’s (1965) placement of *Keichousaurus* in the Keichousauridae within Pachypleurosauroidea (= Pachypleurosauria).

In the 21st century, multiple pachypleurosaur-like taxa were newly discovered from the Early to Middle Triassic of China, which triggered comprehensive analyses of eosauropterygian phylogeny and meanwhile arose disputes on the keichousaurid origin (*e.g.*, Jiang et al., 2008a; Jiang et al., 2008b; Jiang et al., 2008c; Jiang et al., 2014; Wu et al., 2011; Shang, Wu & Li, 2011; Shang, Wu & Li, 2017; Liu et al., 2011; Cheng et al., 2016; Xu et al., 2022; Xu et al., 2023; Hu, Li & Liu, 2024). Some authors (*e.g.*, Holmes, Cheng & Wu, 2008; Shang, Wu & Li, 2011; Liu et al., 2025) removed *Keichousaurus* outside of Pachypleurosauridae and referred it to the Nothosauroidea or to Eusauropterygia. However, other authors referred to additional taxa (*e.g.*, *Dianopachysaurus*) to Keichousauridae and moved the family back into Pachypleurosauroidea again (*e.g.*, Liu et al., 2011; Lin et al., 2021; Xu et al., 2022; Xu et al., 2023). The latter scenario is supported by our analysis. For the first time, four taxa (*Dianopachysaurus*, *Dawazisaurus*, *Panzhousaurus* and *Keichousaurus*) are united here into a monophyletic clade (newly defined Keichousauridae) that is sister to the Pachypleurosauridae, and three other Chinese taxa (*Majiashanosaurus*, *Diandongosaurus* and *Dianmeisaurus*) are nested at the base of Pachypleurosauroidea. This revised scenario would rather support an eastern Tethyan origin for Keichousauridae. As indicated by their body form and depositional environment, keichousaurids would inhabit intraplatform basins and shallow epicontinental marine habitats (Lin & Rieppel, 1998), and they probably lack the ability to undertake trans-Tethyan migrations. That could be the reason that all keichousaurids were endemic to Southwest China. This family has a relatively short geological range from middle Anisian to late Ladinian (∼244–240 Ma), representing a fast radiation of pachypleurosauroid eosauropterygians in the Middle Triassic Yangtze Sea.

## Conclusions

**Figure 9:**
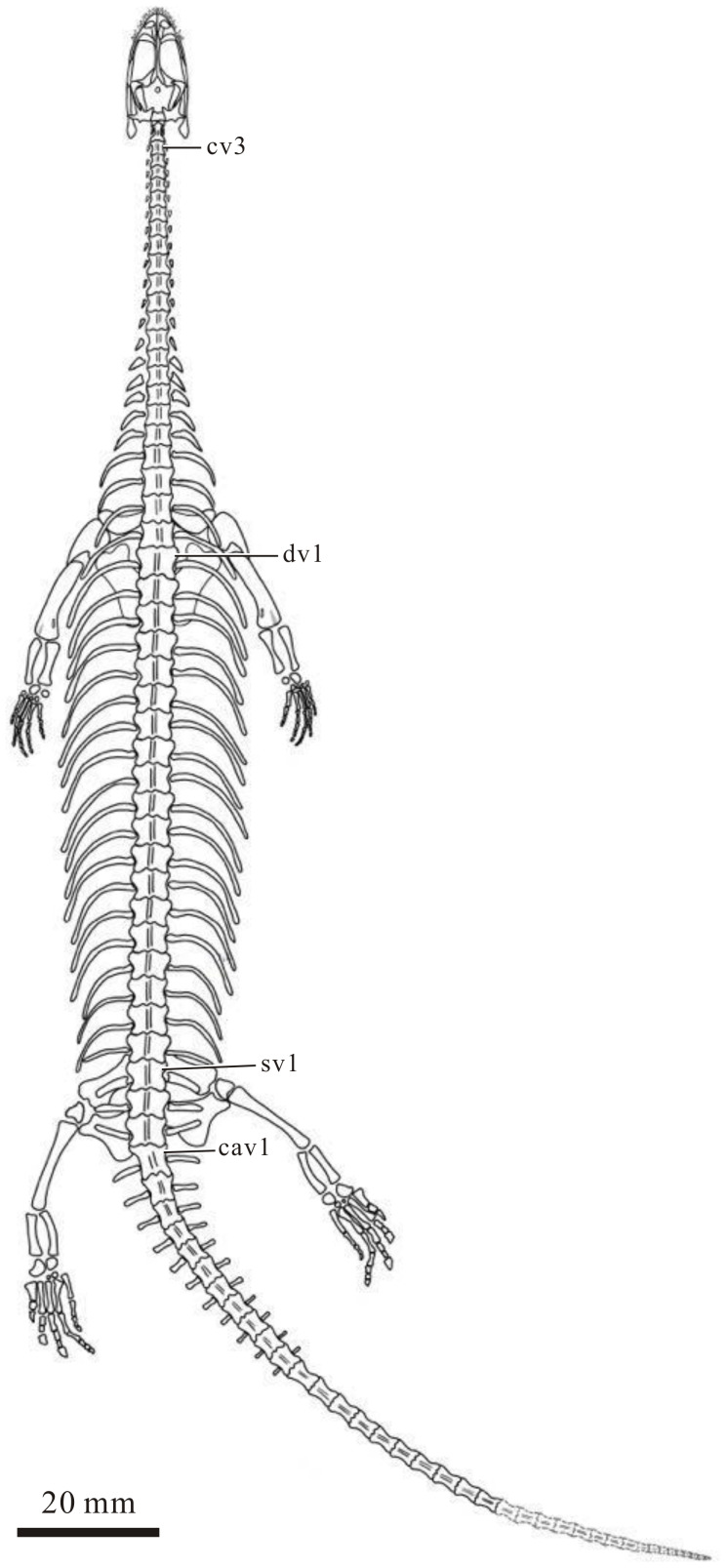
Tentative reconstruction of the skeleton of *Panzhousaurus rotundirostris* in dorsal view. The distal portion of the tail (shown in light grey with dashed lines) is reconstructed based on complete keichousaurid specimens (*e.g.*, *Dawazisaurus*; see text for details).

The discovery of a new, juvenile specimen of *Panzhousaurus rotundirostris* and its comparative study of the holotype and GMPKU-P-3241 stimulate a detailed redescription and revision of this species. The revision accommodates significant changes to the reconstruction of *P. rotundirostris*, including the skull roof, circumorbital bones and jaws (Fig. 4C, Fig. 9). The juvenile status of IVPP V30747 is well confirmed by a series of features (*e.g*., smaller body size, relatively larger orbit, unfused neural arches, and fewer carpal ossifications and caudal ribs). The wealth of new anatomical data for *P. rotundirostris* has facilitated a phylogenetic analysis, the results of which recover *Panzhousaurus* as the sister taxon to *Keichousaurus*, and support an eastern Tethyan origin for Keichousauridae. The diagnoses have been emended for both Pachypleurosauroidea and Keichousauridae. The emended diagnoses of Pachypleurosauroidea and Keichousauridae, along with the revision of Keichousauridae, contribute to a better understanding of the fast radiation of pachypleurosauroid eosauropterygians in the Middle Triassic Yangtze Sea.

## Anatomical abbreviations

an: angular
ana: atlas neural arch
ar: articular
as: astragalus
at: atlas
ati: atlas intercentrum
atr: atlas rib
ax: axis
axr: axis rib
bo: basioccipital
ca: calcaneum
cav1: the 1st caudal vertebra
cl: clavicle
cn: coronoid
co: coracoid
cv3: the 3rd cervical vertebra
cv24: the 24th cervical vertebra
d: dentary
d1: the distal distal carpal I
d3: the distal carpal 3
d4: the distal carpal IV
dr1: the 1st dorsal rib
dt3: the distal tarsal III
dt4: the distal tarsal IV
dv1: the 1st dorsal vertebra
dv19: the 19th dorsal vertebra
dv20: the 20th dorsal vertebra
ec: ectopterygoid
ecg: ectepicondylar groove
en: external naris
enf: entepicondylar foramen
eo-po: exoccipital-opisthotic
f: frontal
fe: femur
fi: fibula
h: humerus
il: ilium
int: intermedium
is: ischium
j: jugal
m: maxilla
mc1: the 1st metacarpal
mc5: the 5th metacarpal
mt1: the 1st metatarsal
mt5: the 5th metatarsal
n: nasal
of: obturator foramen
p: parietal
pa: palatine
pm: premaxilla
po: postorbital
pof: postfrontal
prf: prefrontal
pt: pterygoid
pu: pubis
q: quadrate
qj: quadratojugal
r: radius
sa: surangular
sc: scapula
so: supraoccipital
sp: splenial
sq: squamosal
sr: sacral rib
st: supratemporal
sv1: the 1st sacral vertebra
sv3: the 3rd sacral vertebra
ti: tibia
u: ulna
ul: ulnare

## Additional Information and Declarations

### Data Availability

The following information was supplied regarding data availability: The raw data and code are available at Zenodo: Xu, G.-H. (2026). Supplementary figures, character list, data matrix (TNT and PAUP), and TNT phylogenetic results (EW and EIW) [Data set]. Zenodo. https://doi.org/10.5281/zenodo.18871621.
